# Psychosis and Psychotic-Like Symptoms Affect Cognitive Abilities but Not Motivation in a Foraging Task

**DOI:** 10.3389/fpsyg.2020.01632

**Published:** 2020-07-31

**Authors:** Wenche ten Velden Hegelstad, Isabel Kreis, Håkon Tjelmeland, Gerit Pfuhl

**Affiliations:** ^1^TIPS Centre for Clinical Research in Psychosis, Psychiatric Division, Stavanger University Hospital, Stavanger, Norway; ^2^Department of Psychology, UiT – The Arctic University of Norway, Tromsø, Norway; ^3^Department of Mathematical Sciences, Norwegian University of Science and Technology, Trondheim, Norway

**Keywords:** metacognition, goal-directed behavior, schizophrenia, short-term memory, decision-making

## Abstract

**Background and Objective:**

Goal-directed behavior is a central feature of human functioning. It requires goal appraisal and implicit cost-benefit analyses, i.e., how much effort to invest in the pursuit of a certain goal, against its value and a confidence judgment regarding the chance of attainment. Persons with severe mental illness such as psychosis often struggle with reaching goals. Cognitive deficits, positive symptoms restricting balanced judgment, and negative symptoms such as anhedonia and avolition may compromise goal attainment. The objective of this study was to investigate to what degree symptom severity is related to cognitive abilities, metacognition, and effort-based decision-making in a visual search task.

**Methods:**

Two studies were conducted: study 1: *N* = 52 (healthy controls), and study 2: *N* = 46 (23 patients with psychosis/23 matched healthy controls). Symptoms were measured by the CAPE-42 (study 1) and the PANSS (study 2). By using a visual search task, we concomitantly measured (a) accuracy in short-term memory, (b) perceived accuracy by participants making a capture area or confidence interval, and (c) effort by measuring how long one searched for the target. Perseverance was assessed in trials in which the target was omitted and search had to be abandoned.

**Results:**

Higher levels of positive symptoms, and having a diagnosis of psychosis, were associated with larger errors in memory. Participants adjusted both their capture area and their search investment to the error of their memory. Perseverance was associated with negative symptoms in study 1 but not in study 2.

**Conclusion:**

By simultaneously assessing error and confidence in one’s memory, as well as effort in search, we found that memory was affected by positive, not negative, symptoms in healthy controls, and was reduced in patients with psychosis. However, impaired memory did not concur with overconfidence or less effort in search, i.e., goal directed behavior was unrelated to symptoms or diagnosis. Metacognition and motivation were neither affected by cognitive abilities nor by negative symptoms. Clinically, this could indicate that struggles with goal directed behavior in psychosis may not solely be dependent on primary illness factors.

## Introduction

Motivation and goal-directed behaviors are complex phenomena. Consider the following: You meet four students and all tell you that they are motivated to pass an important exam. Student A is very smart, she knows she is high performing, and she spends some time but not all day on studying for the exam. Student B is smart, and she knows she can pass the exam if she studies all day. Student C is not as smart as students A and B, but she still wants to pass the exam and she, too, studies all day. Student D is as smart as student C but thinks of herself as being as smart as student A and does not spend all day studying for the exam. This example illustrates the interplay of cognitive ability, knowing about one’s ability, and effort (here the amount of learning spent) affecting goal-directed behavior. Without asking about motivation, only measuring the outcome or the effort, we would draw different conclusions. Students A and D would appear as not having spent much effort, hence not being motivated. Students C and D may appear not motivated as they might not have passed the exam.

We often infer motivation from the intensity, e.g., speed or vigor, with which a person tries to achieve a goal and how much effort she invests in a task, respectively. However, this equates motivation with effort, while effort depends on knowing one’s abilities and chances of reaching the goal. For physical effort this is obvious: A well-trained person needs less energy to catch the last bus into town than an untrained person, but both can be similarly motivated. A person using crutches may also be similarly motivated to catch the last bus but knows they cannot make it and hence is not running to the bus. This knowing of what one can achieve requires metacognition (Norman et al., 2019).

Goal-directed behavior, which is an operationalization of motivation (Hebb, 1955; Duffy, 1957), requires knowledge about one’s abilities (metacognition), and the effort needed for reaching the goal. The latter can be, for example, how fast (latencies, vigor) or how hard (perseverance) one tries to reach the goal (Salamone and Correa, 2012). Conversely, if one overcomes the costs of effortful actions to achieve a desired outcome (Chong et al., 2016), the costs will depend on one’s abilities. It is rational to not spend any effort on a too costly or fruitless task (Pfuhl et al., 2009), or alternatively, to try solving the task in a cheaper manner (Pfuhl, 2012; Mækelæ et al., 2018). Thus, to infer about a person’s motivation, we have to know the relative effort spent. Only measuring absolute effort spent does not suffice.

Amotivation and effort-related impairments are common symptoms in many mental disorders, including schizophrenia (Fervaha et al., 2015) and depression (Clery-Melin et al., 2011). Both are associated with a lack of goal-directed behavior, impeding daily functioning (Barch et al., 2014; Bergé et al., 2018). However, effort-related impairments are sensitive to the tasks in question (Horan et al., 2015; Reddy et al., 2015). Often tasks used in research or clinical practice do not control for cognitive abilities and metacognition, which is known to also be affected in these mental disorders (Moritz et al., 2015; Sun et al., 2017; Norman et al., 2019). Indeed, if a person is like student D, then it is metacognition, not effort spending, that is aberrant. Students C and D need in absolute terms to spend more effort.

To tease apart which factors contribute to motivation and goal-directed behavior, we developed a foraging task that simultaneously measures how good a person’s visual short-term memory is, how good a person thinks their memory is (metacognition), and, consequently, how much effort they spend in finding a predefined target. By using a mathematical model, we can also infer the costs of foraging and thereby calculate the relative effort spent. We tested this in two studies. In study 1 we recruited healthy participants from the general population that varied in the severity of dysphoria and psychotic-like experiences. We thereby aimed to have a larger variation in cognitive abilities and metacognitive abilities than found in a pure student sample. In study 2 we recruited patients with a diagnosis of schizophrenia and related psychoses, as there is inconsistency in the literature (Gold et al., 2015; Green et al., 2015; McCarthy et al., 2016; Culbreth et al., 2018) in whether they lack effort or not, and whether this is due to cognitive dysfunctions, aberrant metacognition, or true amotivation, i.e., no desire to reach the goal. In both studies we used the same paradigm to measure concomitantly cognitive ability, here visual short-term memory, implicit metacognition (Honig et al., 2020), and effort in a foraging task (Pfuhl et al., 2009, 2013). We first present the task before we review relevant clinical literature.

### The Precision and Motivation Task – A Simple Foraging Task

In an attempt to disentangle the various components inherent in tasks involving goal-directed behavior, we developed the Precision and Motivation Task (PMT). The task is based on a mathematical model (Pfuhl et al., 2009), trying to quantify the question of how much effort one should invest in an activity (effort estimation), and when to abandon it in relation to how likely it is to find reward relative to the cost of searching (reward valuation and memory estimation).

To illustrate, imagine a treasure hunt where you hide a small cache with sweets for a children’s party somewhere in a nearby park in the morning. At lunch time you check on the cache and find it again. In the late afternoon you (with the children) go out to find the cache. Since you are distracted on your way to the location you have a hard time finding the exact location. You don’t want to embarrass the children and continue searching but you also see families with dogs, and after a while consider the option that the cache got raided and abandon the treasure hunt.

In between the extremes of knowing for sure and having no idea, there is an optimal limit of investing in the search. As you forget (memory of the location of the cache becomes less precise), the optimal search limit first rises, but then it declines steeply. Furthermore, your investment depends also on the probability of it being there, and not having been removed by a third party (here: cache raided by a dog). This probability of a third party is never zero. However, the closer it is to zero, the longer you should search irrespective of the precision of your memory. If the probability of the target being gone is high, you should–as in the case of having poor memory–not start searching at all (Pfuhl et al., 2009). This cognitive weighing of pros and cons of investing effort is an example of effort-based decision making.

In this foraging task the investment in search depends on how well one thinks one’s memory is, and how likely one thinks that no third party raided the cache. In our task, we inform about the probability of the cache being raided, and measure directly how well a person thinks she remembers the cache. We refer to how well one thinks one remembers as meta-cognitive ability in the remainder of the article.

In sum, this task allows us to measure how precise a person’s memories for a target actually are (visual short-term memory), how precise they believe those memories are (meta-cognitive ability), and how much effort they invest in searching for the target, respectively (Pfuhl et al., 2013). We also measured perseverance, latencies, and vigor. Vigor has been found to be an implicit measure for the subjective utility of the outcome (Shadmehr et al., 2019). Perseverance is the duration of search relative to one’s metacognitive ability.

### Motivation, Vigor, and Effort-Based Decision Making in Psychosis and Schizophrenia

It has previously been thought that motivational deficits in schizophrenia were linked to the anhedonia and the blunting of affect seen in negative symptoms (Rømer Thomsen et al., 2015). However, it appears that in-the-moment hedonic experiences are in fact intact (Llerena et al., 2012). This has led researchers to explore other possibilities, now suggesting that individuals with negative symptoms have deficits in a range of reward-related processes, making it difficult to translate reward information into motivated behavior (Blanchard and Cohen, 2006; Whitton et al., 2015; Barch et al., 2016). This has been proposed as a deficit of vigor, the speed of activities toward a goal, dependent on the computation of reward expectation, i.e., subjective goodness of an option, and effort (Shadmehr et al., 2019).

Others have linked negative and depressive symptoms, both prevalent in schizophrenia (an der Heiden et al., 2016) to amotivation (Pelizza and Ferrari, 2009), and accordingly to a lack of goal-directed behavior (Brown and Pluck, 2000). A range of studies have found that individuals with psychotic disorders or schizophrenia have effort-related impairments (Barch et al., 2014; Whitton et al., 2015) and that there is an association between the degree of negative symptoms and these impairments (Bergé et al., 2018). In healthy populations similar findings have been reported for negative symptom-like phenomena (Stefanis et al., 2002; Terenzi et al., 2019).

### Cognitive Abilities in Psychosis and Schizophrenia

Research on cognitive dysfunctions in psychosis in general, and schizophrenia in particular, is abundant, as these have been considered core features for at least the last century (Mesholam-Gately et al., 2009). They have been studied in relation to negative symptoms, which are characterized by a lack of interest in goal-directed behavior and affective expression (Andreasen et al., 1995), as well as psychomotor poverty (Liddle and Barnes, 1990), and in relation to positive symptoms (Moritz et al., 2008), characterized by unwilled mental experiences such as hallucinations or paranoia. They are known to appear before psychosis onset (Barragan et al., 2011; Bora and Murray, 2014) and have also been found in first-degree relatives of persons with psychosis (Snitz et al., 2006; Montag et al., 2012).

Deficits are most prominent in memory (working memory, verbal, and visual memory), processing speed, and visuospatial abilities (Mesholam-Gately et al., 2009). Severity of general symptom load appears to be related to severity of general cognitive deficits in some studies (Barder et al., 2013), but minimally so in others (Dominguez Mde et al., 2009). Negative symptoms specifically appear to be associated with poorer memory (both verbal and visual), verbal fluency and executive functions (O’Leary et al., 2000), and with poorer motor and information processing speed (Rund et al., 2016), or movement vigor. However, some researchers have proposed that it might not be negative symptoms in themselves that drive cognitive dysfunction–or the other way around–but that this relation is moderated by psychological factors such as a defeatist belief (Grant and Beck, 2009) and an underconfidence in one’s abilities (Szu-Ting Fu et al., 2012). The observed overlap of negative with depressive symptoms (an der Heiden et al., 2016) supports this. Both the memory dimension of deficits and negative symptoms are particularly important for daily life functioning, and therefore, highly relevant foci of study (Fu et al., 2017). Both negative symptoms and cognitive deficits are longitudinally more stable compared to positive symptoms, which are more likely to wax and wane (Harvey et al., 2006), leading to the widely held belief that these are the more trait-like core characteristics of schizophrenia.

In sum, motivation, effort-based decision making, and goal-directed behavior in psychosis and schizophrenia appear to be linked through cognitive (working memory, processing speed), vigor (the reflection of the economic evaluation of cost vs. benefit as speed toward a goal), and symptom (especially negative) factors. Several paradigms have been developed to be able to more objectively measure motivation and effort (Gorissen et al., 2005; Horan et al., 2015; Reddy et al., 2015; Bergé et al., 2018). These tasks measure how much effort one is willing to exert across different reward amounts and reward probability conditions. They do not measure effort relative to one’s cognitive abilities and belief in one’s abilities. Hence, analyzing the various components involved remains a challenge.

In two studies we investigated the role of the various components in our PMT. Our aim is identifying which component(s) contributes to effort-based decision-making in psychosis. In detail:

As **hypothesis 1a**) we predicted neurocognitive deficits in participants to be associated with load of symptoms and symptom-like phenomena. In **hypothesis 1b**) we predicted a difference in metacognitive ability among individuals: On the one hand we expected dysphoric participants to be underconfident (Szu-Ting Fu et al., 2012) and participants with psychotic-like experiences to be overconfident about their memory (Moritz et al., 2015).

In **hypothesis 2a)** we expected that participants with many symptoms will search less than needed to find the target. In **hypothesis 2b)** we expected reduced speed and vigor among participants with predominantly negative symptoms and symptom-like phenomena and dysphoria compared to participants scoring predominantly high on positive symptoms and symptom-like phenomena and to the control group with no symptoms.

## Study 1 – Investigating Goal-Directed Behavior Along the Psychosis Spectrum

The aim of this study was to identify which of the several psychosis-like phenomena contribute the most to a lack of motivation and goal-directed behavior in a non-clinical sample. We aimed to recruit healthy, not at risk, participants with a first-degree relative diagnosed with a psychotic disorder to increase the odds that our sample varies in the severity of psychosis-like symptoms.

## Participants

Fifty-three individuals with no current or prior history of mental illness were recruited through social media and on a volunteer basis. Twelve of the participants had first-degree relatives with mental illness history, of whom four reported a relative with a schizophrenia diagnosis and eight with a first-degree relative with a bipolar diagnosis. The age ranged from 18 to 49 years, with mean age of 26 (*SD* = 7.1). Thirty-seven (70%) of the participants were women. Participants were excluded if they presented a substance use disorder (except nicotine), clinically significant psychiatric symptoms, or if they had neurological disorders. One participant was excluded due to abandoning testing after two tasks, leaving 52 participants.

Participation was rewarded with a gift certificate worth 150 NOK, approximately $16.

### Materials

#### Neurocognitive Tests

We used the Trail Making Test (TMT) A and B (Reitan and Wolfson, 1985). TMT A yields an indication of psychomotor speed and visual processing, which is an attention and processing speed measure, whereas TMT B is an estimate of mental flexibility and inhibition, central compounds of cognitive control and executive function. The task was administered and interpreted as described in Bowie and Harvey (2006). We used the Digit Symbol Substitution Task (DSST), a test that is sensitive to psychomotor speed and general speed of information processing. The score is computed by counting the number of correct pairings completed in 90 s.

#### Precision and Motivation Task

We developed an effort-based decision-making paradigm, where one has to search for a previously seen shape (visual short-term memory), indicate how well one thinks that one remembers the shape (implicit metacognitive assessment), and decide to search for it with the probability of succeeding in finding the shape signaled (effort-based decision). There are points to be scored for correct responses. We presented the task as a computer game with a background story of squirrels hiding nuts and other squirrels stealing them. The game has four stages.

**FIGURE 1:**
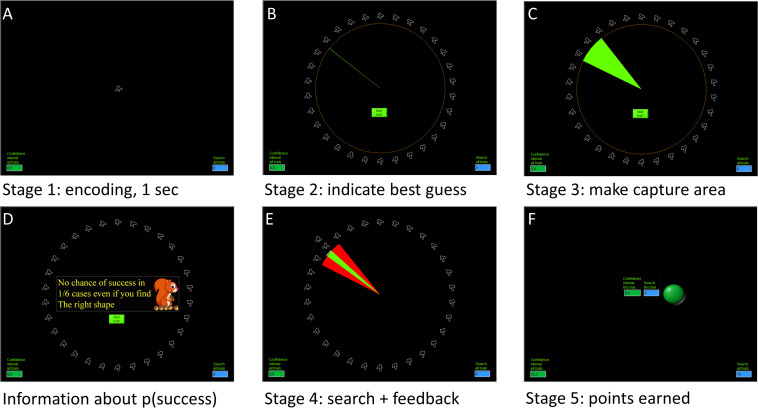
Precision and motivation task. **(A)** Sample phase, here a squiggly shape is shown for 2 s. **(B)** Retrieval phase, 30 similar shapes are organized in a circle. The participant indicates the location of the shape looking most alike as the one seen in the sample phase. **(C)** Confidence phase, participants make a capture area large enough to be certain that the shape from the sample phase is included in the area. **(D)** The chances of reward are signaled. The probability of not finding the “nut” is indicated by the squirrel, either 1/6 or 1/3. **(E)** Search phase, the participant searches for the “nut,” starting at the indicated location from the retrieval phase. **(A)** White line stretches in both directions for each click, indicating the search. When found or abandoned, the program provides visual feedback on the capture area with red indicating excess area. **(F)** Points earned for making a well-calibrated capture area (shown in the green rectangle), and points earned for search (shown in the blue rectangle) are presented. Total earnings are always presented in the bottom, bottom left for capture area and bottom right for search. Apart from the sample phase, all other phases were self-paced. Abandoning retrieval, confidence judgment, and search was possible by clicking on the “next trial” button (no points earned).

*Stage 1*: An abstract shape (the “nut”) is shown for 2 s (Figure 1A). The participant is instructed to remember the shape.

*Stage 2*: The participant must indicate where, among 30 similar shapes arranged continuously on a circle, the previously seen shape is located. If she does not remember, she can move on by clicking the “next trial” button. This stage measures the error of one’s memory as the deviation between the chosen shape and the target shape, measured in degrees (Figure 1B).

*Stage 3*: From the indicated location of the shape the participant draws a capture area (Figure 1C). The participant is instructed to make the area large enough so that she is sure the shape is located somewhere inside the capture area (Graf et al., 2005; Pfuhl et al., 2013; Honig et al., 2020). The size of the capture area can be considered an implicit measure of confidence. Points are received depending on the size of the capture area in relation to one’s error in memory. The maximum score is 10 points in each trial, with points subtracted if the capture area is made too large, but no points earned if the capture area is made too small. The points are presented after each trial (stage 5). Metacognitive error is calculated as the ratio of the logs of the size of the capture area and the memory (stage 2). A ratio of 1 indicates perfect calibration between real and perceived precision of memory. A ratio larger than 1 indicates underconfidence whereas a ratio smaller than 1 indicates overconfidence.

*Stage 4:* The participant searches for the shape after being presented with information about the probability of finding it, represented by a mean or a kind squirrel (Figure 1D). The mean squirrel is hungry and steals the nut in 1 out of 3 cases. The kind squirrel is less hungry and steals the nut in 1 out of 6 cases (representing 67 and 83% probability of success or finding the nut). The participant was informed that the nut will be found if searched long enough but that search has to be abandoned if she thinks the nut got stolen. These stolen trials are referred to as no-target trials and are not signaled. Search was done by clicking the left mouse key repeatedly. The search started where the participant indicated she thinks the nut is located (same as for stage 3) and expanded equally to each side (Figure 1E). The search ended either when the search radius reached the location of the nut in all but no-target trials, or the participant abandoned search by clicking “next trial.” Ten points were received if the nut was found, otherwise the search yielded zero points. Hence, in each trial, participants earned points for their metacognitive ability (stage 3, point amount variable) and success (10 or 0 points). The search phase represents goal-directed behavior and has to be seen in relation to the capture area. Searching less than the indicated capture area means that one spends less effort than one’s belief or (meta-) memory indicates. Accordingly, we calculated a perseverance score as the ratio of logs of search radius in no-target trials and the size of the capture area. A number larger than 1 means that participants searched longer than indicated by the capture area made. Conversely, a score below 1 indicates searching less than announced by the capture area made.

*Stage 5:* presented the earned points, i.e., feedback on how many points participants earned in stage 3 for making a capture area, where the maximum was 10 points and points less than 0 (negative points) were possible if an excessive large capture area was made, and in stage 4 where one either received 10 points or no points (Figure 1F). There was no incentive for abandoning a trial.

The PMT had 45 trials, 30 trials with 1/6 probability of the nut being stolen, 15 trials with 1/3 probability of the nut being stolen. In stolen (no target) trials one sees the maximal effort a participant is willing to exert for a reward. The task was programmed in Labview.

The task measured precision in visual short-term memory, perceived precision, hit rate, absolute and relative search effort, latencies and vigor by which participants searched.

#### CAPE-42

To measure subclinical symptoms, the participants completed the Norwegian version of the Community Assessment of Psychic Experiences (CAPE-42) (Stefanis et al., 2002) questionnaire. The CAPE-42 was developed to measure the lifetime prevalence of psychotic experiences in the general population. The CAPE has three subscales: positive (CAPE-P), negative (CAPE-N) and depressive (CAPE-D). The questionnaire was implemented in Qualtrics.

Internal consistency for the CAPE-42 total score (α = 0.94), as well as for the positive symptom subscale (α = 0.84), the negative symptom subscale (α = 0.90), and the depressive symptom subscale (α = 0.87), were high. CAPE negative and CAPE depressive subscale correlated highly, ρ = 0.823, *p* < 0.001. Because of this correlation and the significant conceptual overlap (an der Heiden et al., 2016), and to maximize the probability of score variability in a healthy population, we created a new subscale CAPE-ND being the sum of those two subscales.

### Procedure

Participants read and signed the consent form and completed a survey asking about education and employment, alcohol habits, medication and substance use habits, mental health, and neurological disorders. Thereafter they performed the TMT A and B and the DSST. These tests were done with pen and paper and a stopwatch. Next, we demonstrated the PMT introducing the stages and point structure step-wise in six demonstration trials. The task took approximately 22 min to complete. Lastly, participants answered the CAPE-42.

### Ethics

The project was approved by the Regional Committee for Medical and Health Research Ethics Norway, Region West (2011/1198/REK Vest).

### Statistical Analyses

We used multiple regression for TMT A, TMT B, DSST with CAPE-P, CAPE-ND, and age as predictors. The results do not change when including education (Tombaugh, 2004).

For the PMT we calculated the average latency to start a trial and vigor (speed at which the search clicks were made). Average error in memory was the absolute error of memory in all 45 trials. Similarly, the average size of the capture area was based on all trials where a participant made a capture area. Metacognitive error was the relative size of the capture area to one’s error in memory.

Search radius was calculated separately for trials with a high or low probability of finding the target, and for trials where the target could be found, and where it could not be found. In trials where the shape could not be found we calculated the perseverance score, which expressed search investment relative to perceived error. Hits are all trials where the search was long enough to include the target shape, and a high hit rate suggested a decision criterion based on low effort sensitivity.

Finally, we calculated the costs of searching by using the 1D approach described in Pfuhl et al. (2009). The cost function depends on one’s perceived error and search as well as the probability of success. Details of the mathematical derivation and code to evaluate the costs of searching can be found here: https://osf.io/9bfxt/.

We used multiple regression for the indices of the PMT with the CAPE-P, CAPE-ND, and age as predictors. Data was analyzed in R and JASP (JASP Team, 2020).

### Results

None of the participants had psychiatric symptoms indicative of mental disorder; however, 13 participants reported feeling depressed or dysphoric at a sub-clinical level.

**Hypothesis 1a** predicted that neurocognition as measured by the TMT A and B, the DSST and error in the visual short-term memory task would be impaired in participants with high CAPE-42 scores. Table 1 provides the descriptives for those four tests, as well as for the symptom severity in this sample.

**TABLE 1:** Symptom severity and neurocognitive task performance (*N* = 52) in study 1.

	M(*SD*)	Minimum	Maximum
CAPE-P	25.62 (4.9)	20.00	41.00
CAPE-ND	42.64 (11.1)	26.00	69.00
TMT-A	24.74 (5.9)	14.37	38.18
TMT-B	59.16 (16.6)	30.49	100.85
DSST	57.48 (7.9)	37.00	73.00
Error in PMT	18.29 (7.3)	7.23	40.47

*CAPE-P, positive symptom scale from the CAPE-42. CAPE-ND, negative/depressive symptom scale. TMT A and TMT B is measured in seconds, DSST is measured as number of correct symbols. Error in PMT is measured in degrees. Means (*M*; with standard deviations *SD*).*

There was no significant relationship between TMT A and CAPE-P (β = 0.18, *t* = 1.07, *p* = 0.29) or CAPE-ND (β = −0.07, *t* = −0.42, *p* = 0.68), but there was a significant relationship of age and TMT A (β = 0.50, *t* = 3.83, *p* < 0.001), i.e., younger people were faster. There was a significant relationship of TMT B performance with lower levels of CAPE-P (β = 0.39, *t* = 2.09, *p* = 0.042), but not with CAPE-ND (β = −0.22, *t* = −1.23, *p* = 0.22) or age (β = 0.13, *t* = 0.91, *p* = 0.37).

There was a significant negative relationship between CAPE-P and completed pairings in DSST (β = −0.53, *t* = −3.29, *p* = 0.002) and between age and DSST (β = −0.39, *t* = −3.10, *p* = 0.003), i.e., more positive symptoms and older age were associated with fewer completed pairings. There was no significant relationship between DSST and CAPE-ND (β = 0.08, *t* = 0.52, *p* = 0.60).

The error in memory was statistically significantly associated with CAPE-P (β = 0.672, *t* = 4.406, *p* < 0.001), i.e., the more positive symptoms, the poorer visual short-term memory. There was no significant relationship between visual short-term memory and CAPE-ND (β = −0.227, *t* = −1.517, *p* = 0.136) or age (β = 0.141, *t* = 1.141, *p* = 0.259).

**Hypothesis 1b** predicted aberrant metacognition in participants scoring high on symptoms, i.e., participants reporting positive symptom-like experiences would be overconfident whereas participants high on depression/negative symptoms would be underconfident.

#### Metacognitive Error

On average participants were well calibrated, i.e., the size of the capture area corresponded well to their error, *M* = 1.037, *SD* = 0.122. The predictors explained only 11.4% of the variance, *F* = 2.053, *p* = 0.119. The mean *hit rate*, searching long enough to find the target, was 0.66 (*SD* = 0.11), ranging from 0.42 to 0.96. The predictors explained only 11% of the variance, *F* = 1.977, *p* = 0.13.

Thus, we found that symptom severity and age did not relate to how well people assess their memory to be; there was no clear indication of over- or underconfidence, i.e., aberrant metacognition, in participants with psychotic-like experiences or those with dysphoria.

**Hypothesis 2a** predicted that goal-directed behavior would be diminished in people scoring high on negative or depressive symptoms, i.e., that they would search less than indicated by the capture area. Since the search radius depends on how well a person thinks she remembers the shape, we used the perseverance score. A score larger than 1 indicates searching beyond the capture area made. In the high probability condition the perseverance score was 1.24 (*SD* = 0.166), and in the low probability condition the perseverance score was 1.191 (*SD* = 0.168), indicating that participants searched beyond the capture area made in both the high and the low probability conditions. One sample *t*-test confirmed that the search exceeded the capture area made [high probability condition: *t*(51) = 10.4, *p* < 0.001, *d* = 1.443; low probability condition: *t*(51) = 8.21, *p* < 0.001, *d* = 1.138]. Perseverance was lower in the low probability condition, *t*(51) = 2.968, *p* = 0.005, *d* = 0.412 than in the high probability condition. Next, we performed stepwise multiple regressions with positive and negative/depressive symptom scores and age as predictors. In the high probability condition, the remaining predictor was the CAPE-P, β = −0.305, *t* = −2.264, *p* = 0.028, indicating less perseverance associated with higher positive symptom scores. In contrast, for the low probability condition, only the CAPE-ND remained, β = −0.324, *t* = −2.417, *p* = 0.019, indicating less perseverance for higher negative/depressive symptoms. Since CAPE-P and CAPE-ND correlated highly (ρ = 0.616, *p* < 0.001), general symptom severity (total CAPE-42 score) related to search investment, i.e., the more symptoms a participant reported, the less was their relative search investment.

**Hypothesis 2b** predicted that individuals with many negative or depressive symptoms show longer latencies and lower vigor. The average latency to start a trial was 2.76 s (*SD* = 1.14), ranging from 1.18 to 6.26. The average vigor in high probability trials was 0.25 (*SD* = 0.09), ranging from 0.15 to 0.64. In the low probability trials the mean vigor was 0.26 (*SD* = 0.07), ranging from 0.16 to 0.59. That is on average a participant made four clicks per second. There was no relationship between latency, vigor, and symptom severity or age, all *p* > 0.05.

**FIGURE 2:**
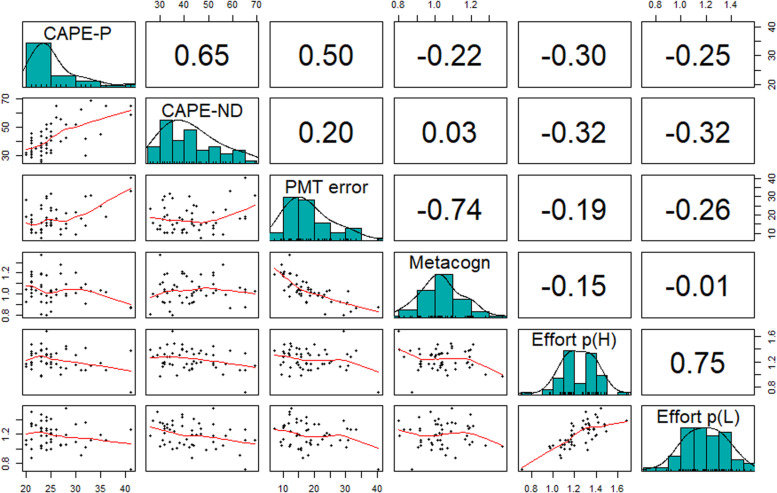
Association of symptom severity with error in memory (deviation), metacognitive error, and investment in search in study 1. Numbers represent Pearson’s correlation coefficients, and asterisks indicate a *p*-value below 0.05. The more positive symptoms the more errors in memory (Pearson’s *r* = 0.5, *p* < 0.001) and also the more negative/depressive symptoms (*r* = –0.65, *p* < 0.001). The more negative/depressive symptoms a participant had, the less the person invested in searching relative to the belief in their memory (*r* = –0.32 *p* = 0.02). General symptom severity (CAPE-P and CAPE-ND) related to relative effort in both probability conditions (not shown). Perseverance (effort) in the high and low probability condition were highly correlated (*r* = 0.75, *p* < 0.001). Finally, the worse the memory (larger PMT error) the more overconfident participants were (*r* = –0.74, *p* < 0.001).

Finally, we calculated the costs of searching and found that it did not relate to symptom severity or age, *p* > 0.4. Figure 2 summarizes the relationship between the CAPE-42 subscores and error in memory, perceived error in memory, and relative investment in search (perseverance).

## Study 2 – Assessing Goal-Directed Behavior in Patients With Schizophrenia or Related Psychosis

Study 1 recruited participants along the psychosis continuum. Cognitive abilities were lower among those with more symptoms, and symptom severity was associated with diminished effort spent, despite similar costs.

We therefore predicted that: **H1a**: Patients perform worse on the neurocognitive tests compared to matched controls. **H1b**: Patients have an aberrant meta-cognitive ability compared to healthy controls. **H2a**: Goal-directed behavior is reduced, i.e., search is less than indicated by the capture area, in patients compared to controls, and is associated with negative symptoms. **H2b**: Latency and vigor is reduced in patients compared to controls, and is associated with negative symptoms.

## Participants

Study participants (*N* = 23) for the clinical group were recruited from the on-going TIPS 2 (early Treatment and Intervention in Psychosis, start in 2002) study, and invited to partake in this sub-study by one of the research clinical team members, which consists of highly trained psychiatric nurses. Recruitment was conducted between 2015 and 2019, and assessments consisted of the standard TIPS protocol described elsewhere (Melle et al., 2004), with additional assessments as described above. The main inclusion criteria, described in detail elsewhere, were having a first episode of non-affective or affective mood incongruent, non-organic psychosis, and age between 15 and 65 years. Main exclusion criteria were suffering from neurological disorder, primary substance use disorder, or IQ below 70. The patients agreed to baseline assessment, and follow-up after 3 months and 1, 2, and 5 years. Mean duration since inclusion and baseline assessment was 4 years (minimum: 1 year, maximum: 5 years). We recruited 23 age and gender matched participants as healthy controls. One recruited participant had a first-degree relative with psychosis and was excluded. We replaced this participant with one healthy control recruited in study 1. Inclusion criteria for healthy controls were no first-degree relative with a mental health diagnosis, no substance use disorder (except nicotine), no clinically significant psychiatric symptoms, and no neurological disorders. All participants gave informed written consent.

### Materials

We used the same neurocognitive tests and the same PMT as in study 1. Symptoms were assessed by using the Positive and Negative Syndromes in Schizophrenia interview (PANSS) (Kay et al., 1987). The interview, the neurocognitive tests, and the computer task were conducted by three psychologists, under supervision from author WH. The TIPS team holds regular reliability trainings to avoid drift, and previous reliability assessments have proven good reliability (Hegelstad et al., 2012). Patients only received the PANSS.

### Procedure

Patients were first interviewed with the PANSS before starting the behavioral tasks. Matched control participants were not interviewed, however, demographics were recorded (age, gender, education, medication, and handedness).

### Ethics

This study was approved by the Regional Committee for Medical and Health Research Ethics Norway, Region West (2011/1198/REK Vest).

### Statistical Analyses

The indices are similarly calculated as in study 1. Group comparisons are done with *t*-tests, test of equality of variances done with Levene’s test, and effect of symptom severity on outcomes assessed with regression analysis.

### Results

Sample characteristics of study 2 are summarized in Table 2. **Hypothesis 1a** was confirmed: The patient group was significantly slower on the TMT A and TMT B, and they completed fewer pairings in the DSST and had larger errors in memory. Patients needed on average 30.83 s (*SD* = 8.7) on the TMT A, and 98.1 s (*SD* = 45.1) on the TMT B, whereas controls needed 23.24 s (*SD* = 4.8) and 56.88 s (*SD* = 14.45), respectively. Patients solved on average 55.4 (*SD* = 15.1) pairings, and controls solved on average 65.35 (*SD* = 16.3) on the DSST. Patients had larger errors of their visual short-term memory in the PMT. The mean deviation in degrees for patients was 27.5 (*SD* = 11.18), and for controls 22.12 (*SD* = 10.01). Since we had directional predictions we used one-sided Welch’s *t*-test, and all comparisons were statistically significant (Table 3). There was a significant larger variation in the patient group for the TMT A (*F* = 9.13, *p* = 0.004) and TMT B (*F* = 9.909, *p* = 0.003) but not for the DSST or error in memory.

**TABLE 2:** Sample characteristics study 2.

	Patients (*n* = 23)		HC (*n* = 23)	
	N (%)		N (%)	
Gender, female	8 (34.7)		7 (30.4)	
Gender, male	15 (65.3)		16 (69.6)	
**Diagnosis**				
Schizophrenia spectrum	16 (69.6)		n/a	
Affective psychosis	1 (4.3)		n/a	
Other psychosis^1^	6 (26.1)		n/a	

	***M* (*SD*)**	***Md* (*r*)**	***M* (*SD*)**	***Md* (*r*)**

Age	27.1 (8.7)	24 (16–48)	26.0 (9.9)	23 (15–52)
Years of education after secondary school total score	2.7 (2.8)	3 (0–7)	4.4 (2.8)	4 (2–11)
**PANSS subscale**				
Positive^2^	11.9 (4.7)	12 (7–26)	n/a	n/a
Negative^2^	13.5 (6.7)	11 (7–29)	n/a	n/a
General^3^	25.5 (8.9)	24 (16–54)	n/a	n/a
DDD antipsychotic	0.72 (0.77)	0.66 (0–2.7)	n/a	n/a

*Means (*M*; with standard deviations *SD*) and medians (*Md*; with range *r*) are displayed. ^1^Delusional disorder, Psychosis NOS. ^2^Maximum score 63. ^3^Maximum score 112. DDD, defined daily dosis.*

**TABLE 3:** Independent Samples Welch’s *T*-Test, one-sided testing.

	*t*	*df*	*p*	Cohen’s *d*
TMT A	–3.658	34.288	< 0.001	–1.079
TMT B	–4.177	26.479	< 0.001	–1.232
DSST	2.150	43.776	0.019	0.634
PMT error	–1.717	43.465	0.047	–0.506

Within the patient group we performed step-wise regressions with the three subscales of the PANSS and age as predictors. We found that negative symptoms were associated with lower DSST performance, β = −0.473, *t* = −2.462, *p* = 0.023. For TMT scores and error in memory, neither age nor symptom severity were statistically significant predictors.

**Hypothesis 1b** was not confirmed; there was no difference in meta-cognition (e.g., overconfidence in the patient group), *t*(44) = 1.019, *p* = 0.157, *d* = 0.301. Both groups had a ratio slightly smaller than 1, HC group: *M* = 0.972, *SD* = 0.128, SCZ group: *M* = 0.937, *SD* = 0.106. Metacognitive error was not related to PANSS scores in the patient sample, all *p*-values > 0.2.

The mean *hit rate* was marginally smaller in the SCZ group than the HC group with a medium effect size, *t*(44) = 1.552, *p* = 0.064, *d* = 0.458, where patients found on average 56% (*SD* = 12%) of the targets whereas controls found 61% (*SD* = 13%).

**FIGURE 3:**
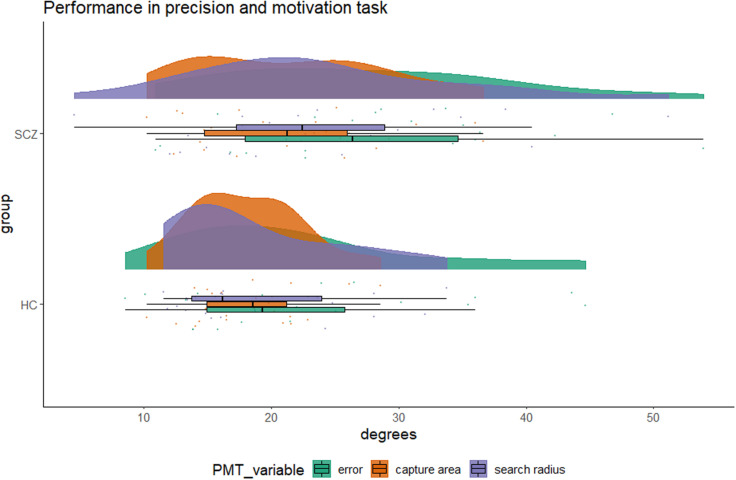
Short-term memory performance expressed as errors in degrees, capture area made (proxy for implicit confidence judgment), and search radii among *n* = 23 patients with a diagnosis of schizophrenia or related psychosis (top row) and *n* = 23 matched healthy controls. The variances between the groups did statistically significantly differ for the capture area made (Levene’s test, *p* = 0.003) but note that we use the capture area relative to error in memory (metacognitive ability) and relative to the search radius in no-target trials (perseverance).

**Hypothesis 2a** was not confirmed. Goal-directed behavior was similar in both groups. We noted, however, that one patient never started searching. The perseverance score was above 1 for both groups and in both conditions, all four *p* < 0.001. Mixed ANOVA yielded less search in the low compared to the high probability condition, *F*(1,43) = 6.653, *p* = 0.013, η*^2^* = 0.015, but no group difference, *F* < 1, or interaction, *F* < 1. Thus, patients and controls alike searched beyond the capture area made and invested more in trials with a higher chance to succeed. We performed a step-wise regression within the patient group. In the high probability condition, age was a statistically significant predictor, *p* = 0.049, but symptom severity did not predict perseverance in high or low probability trials. Finally, we assessed the costs of searching. The two groups had similar search costs, and within the SCZ group search costs did not relate to symptom scores or age, all *p* > 0.1. As can be seen in Figure 3, both groups are similar in their error in memory, capture area made, and search performed.

**Hypothesis 2b** was partly confirmed. There were no group differences for latencies or vigor, all *p*s > 0.1. Within the patient group, step-wise regressions with symptom scores and age as predictors revealed that larger vigor (fewer clicks per minute) was related to higher PANSS general scores in the high probability condition, β = 0.487, *t* = 2.553, *p* = 0.019, but failed to reach statistical significance in the low probability condition (pair-wise: *r* = 0.377).

### Goal-Directed Behavior and Neurocognitive Abilities

After pooling the data from both studies, we explored whether error in memory, metacognitive error, or investment in search and cost of searching are related to the speed in the trail making tasks and the DSST. We also assessed whether perseverance was associated with the error in memory. We used Bonferroni correction (eight tests) and treat *p* < 0.006 as statistically significant.

We found a significant association between speed on the TMT B and error in one’s memory, β = 0.334, *t* = 2.978, *p* = 0.004. TMT-A or DSST were not significant, *p* > 0.2. Metacognitive error, on the other hand, was neither related to TMT-A, TMT-B, nor DSST. These predictors only explained 6.5% of the variance, *F* = 2.165, *p* = 0.097. Goal-directed behavior, as measured with the perseverance score, was not predicted by TMT-A, TMT-B, or DSST, *p* > 0.1. Regarding motor speed, latency to start was positively related with TMT-B, β = 0.258, *t* = 2.264, *p* = 0.026, but not with TMT-A or DSST, *p* > 0.2. Vigor was not predicted by TMT-A, TMT-B, or DSST, *p* > 0.1. Costs of searching were not related to any of the neurocognitive test scores, *p* > 0.1. Finally, there was also no significant association between the perseverance scores (low and high probability condition) and error in memory, *p* > 0.2.

## Discussion

The hypotheses tested in this study were first, that neurocognition be associated with levels of clinical and subclinical symptoms, and second, that positive symptoms be associated with over- and negative/depressive symptoms with underconfidence regarding memory; that third, symptom levels be associated with search effort, and finally, that negative sub-clinical and clinical symptoms be associated with reduced speed and vigor on an experimental task. Neurocognitive deficits were related to psychotic-like experiences and psychosis. Metacognition, expressed as under- or overconfidence, however, was well calibrated irrespective of diagnosis or symptom severity. Search effort and perseverance was more diminished the more psychotic-like experiences one had (study 1) and vigor was more diminished the more general symptoms a patient had (study 2). Goal-directed behavior as measured by the outcome (hit rate) was reduced in patients, but this finding was not statistically significant when compared to healthy controls.

In study 1 we found that neurocognitive deficits, both in the DSST and error in visual short-term memory, were related to positive, and not negative, symptom-like experiences. Similarly, Rossler et al. (2015) found that processing speed in the DSST was related to anomalous perception in healthy adults. Abu-Akel et al. (2016), though, found that participants with predominantly negative and few positive symptoms were less accurate in a visual-spatial working memory task. This difference might be due to our sample consisting of participants that had either very few symptoms (low CAPE-42 score) or had both many negative/depressive symptoms and positive-like experiences.

In study 2, testing participants with a diagnosis of schizophrenia, we replicated previous findings that patients have slower psychomotor speed and visual processing, i.e., have more problems with interference and mental flexibility and also poorer visual short-term memory (Schaefer et al., 2013). The finding that poorer memory performance was associated with lower scores on TMT-B might be explained by the executive component of both tests, in so far that both place a load on working memory and manipulation of “on-line” information. We found some indication that negative symptoms affect performance in the DSST; however, our sample was small and as for study 1, patients often had either few symptoms or both many negative/depressive and positive symptoms. We can therefore not conclude whether positive or negative symptoms contribute more to neurocognitive deficits.

Regarding meta-cognition, here confidence in one’s own memory, we used a non-verbal assessment of metacognition by asking participants to draw a capture area just large enough that it includes the target. This, in our opinion, yielded a more implicit and observable measure compared to that obtained by inquiring about confidence using rating scales. However, this implicit assessment of confidence yielded neither a group difference nor was it related to symptom-like experiences. Indeed, overconfidence seems not to be linked to delusional symptoms (Balzan, 2016). Furthermore, in a motoric-perceptual task Knoblich et al. (2004) found intact automatic self-correction, but a failure to report mismatches in patients. In line with our findings, this suggests that implicit metacognition is intact in psychosis.

Further, we found no overall association between hit rate and symptoms in study 1. In study 2 there was a small to medium effect size with patients having a lower hit rate than controls. Regarding search in those trials where the target could not be found, we found that symptom load was associated with perseverance among healthy participants in study 1, but not in patients. This could perhaps be explained by symptoms above a clinical threshold level no longer exerting marginally higher effects on perseverance. Furthermore, both participants in study 1 and patients in study 2 with higher symptom severity searched either less in relative terms (study 1) or less vigorously (study 2). In study 1, higher symptom levels predicted lower perseverance overall. For positive symptoms, this was more true for the high, and for negative symptoms, more true for the low probability condition, which might be a spurious effect or reflect subjective beliefs in succeeding. One possible explanation might be found in the underconfidence associated with negative/depressive symptoms: No matter the probability, perseverance is lower, while for positive symptoms, perseverance is not compromised by low probability. However, with high probability, search perseverance in positive symptoms might be influenced by hasty decision making, and consequently, less reflection on the actual probability of attaining the goal (Moritz et al., 2017). Still, we caution these results, as the effects are small. Importantly, despite reduced memory, the costs of searching were not different. The cost function takes into account the belief and the actual search investment. It is not a metabolic cost or based on motor behavior solely. Indeed, due to their less precise memory, patients did search longer and had to make more clicks to reach the goal, respectively.

Our results indicate that patients did value reward equally to healthy controls, as we found no reduced vigor or latency (Shadmehr et al., 2019) among the groups. Vigor was shown to reflect the subjective value, not the salience of the outcome. Such a similar subjective evaluation of the outcome agrees with previous research that found intact hedonic experiences (Llerena et al., 2012).

Our results did not support the hypothesized association of reduced effort with negative symptoms of psychosis, and highlight prior inconsistencies in this literature (Gold et al., 2015; McCarthy et al., 2016). This inconsistency may in part be due to the fact that reduced effort is just one of many components that can affect the expression of negative symptoms. Negative symptom expression depends on a range of psychological, behavioral, motor, cognitive, and biological phenomena. Passivity (due to having assumed a patient-role, for instance) resulting in reduced overall effort, or avolition, are only some of them. To illustrate, in the TIPS early detection mental health system it has previously been shown that early intervention and treatment is associated with less severe negative symptoms over the first 5 years of follow-up and superior vocational outcomes after 10 years compared to control areas. This may possibly be linked to intact effort in general, and one can speculate that relatively low levels of negative symptoms also may explain the lack of difference between patients and controls on the effort measure of the task in this study where patients come from the TIPS center. Indeed, in our study presented here, in spite of no association with negative symptoms specifically, patients had significantly slower speeds on the TMT tasks and DSST compared to healthy controls. Follow-up analyses using data from both studies indicated that speed measured by TMT B predicted latency to start search. TMT B also has an executive component, which may also explain this latency, as well as worse performance in the TMT-B correlated with worse performance in the PMT.

Regarding the analogy with the students, our findings suggest that participants with psychotic-like experiences and patients with a schizophrenia or other diagnosis of psychosis are on average more like student C. That is, our results suggest that neither neurocognitive deficits nor symptom patterns alone predicted metacognition or goal-directed behavior. Possible explanations could be that our task assessed metacognition implicitly and was not an obvious effort task. Finally, our data indicate that the relations between symptoms, cognition and meta-cognition are complex and deserving of further study, as the fact remains that many persons struggling with these symptoms do face difficulties investing effort in day-to-day tasks.

## Limitations

The two studies had small sample sizes but still replicated the neurocognitive deficits. Our sample size provided not enough power to detect subtle motivational differences or find subgroups of patients with aberrant motivation. Further, one cannot directly compare symptom-like experiences from the CAPE-42 to PANSS symptoms. However, this could not be avoided since most healthy controls will score below clinical threshold on the PANSS; rendering this instrument not useful in a healthy population. Future studies should use the CAPE-42 also in patients.

## Conclusion

By concomitantly measuring cognitive ability, subjective estimation of cognitive ability, and effort we found similar goal-directed behavior irrespective of symptom severity among persons with psychotic-like experiences and participants diagnosed with psychosis. Implicit metacognition was preserved in psychosis.

## Data Availability Statement

All data as well as the mathematical model of the Precision and Motivation task are available in an Open Science Framework repository: doi: 10.17605/OSF.IO/9BFXT.

## Ethics Statement

The studies involving human participants were reviewed and approved by the Regional Committee for Medical and Health Research Ethics Norway, Region West (in Norwegian: REK Vest). Written informed consent to participate in this study was provided by the participants.

## Author Contributions

GP and WH designed the study and supervised the data collection. GP analyzed the data. HT modeled the cost function. GP, IK, and WH wrote the manuscript. All authors contributed to the article and approved the submitted version.

## Conflict of Interest

The authors declare that the research was conducted in the absence of any commercial or financial relationships that could be construed as a potential conflict of interest.
